# The Mediating Effect of Bicultural Self-Efficacy on Acculturation and Career Decision-Making Self-Efficacy for International Students in South Korea

**DOI:** 10.3389/fpsyg.2022.602117

**Published:** 2022-02-11

**Authors:** Yuan Ying Jin, Sungsik Ahn, Sang Min Lee

**Affiliations:** ^1^Department of Education, Sejong University, Seoul, South Korea; ^2^The Graduate School of Education, Keimyung University, Daegu, South Korea; ^3^Department of Education, Korea University, Seoul, South Korea

**Keywords:** bicultural self-efficacy, acculturation, international student, career development, career counseling

## Abstract

The population of international students in South Korea is growing. During the career development phase, international students face unique challenges related to their bicultural identity and acculturation experiences. The present study examined the role of bicultural self-efficacy on mediating the relationship between acculturation and career decision-making self-efficacy for international students in South Korea. Responses from 120 international students in South Korea were analyzed using structural equation modeling. The results showed that bicultural self-efficacy fully mediated the relationship between acculturation to mainstream culture and career decision-making self-efficacy but did not mediate the relationship between acculturation to heritage culture and career decision-making self-efficacy. The implications for effective educational intervention for international college students’ career development were discussed based on the results.

## Introduction

International students are enrolling in educational institutions in increasing numbers worldwide. Specifically, in South Korea, the population of international students more than doubled in the 4 years between 2015 and 2019 (Korean Ministry of Education, 2019). Recent statistics from the Korean Ministry of Education (2019) show that 60,774 international undergraduate students are enrolled in university in South Korea, which is 2.6% of all higher education students in the country (2,346,674). Given the rapid demographic change on campuses throughout the country, it is important to study this population to facilitate students’ career development.

The increase in the number of international students in Korea is due to two major factors. First, in 2004, the Korean government initiated the “Study Korea” project to improve the global competitiveness of Korean universities by recruiting international students. The government then initiated a new “Study Korea” project in 2012 and announced that Korean universities would aim to recruit 200,000 international students by 2020, although the time frame was later extended to 2023 (Korean University Newspaper, 2015). Second, in tandem with the government initiative, some universities in South Korea expanded their efforts to recruit international students in order to compensate for the loss of income that had resulted from an inability to meet their minimum recruitment quotas, which in turn had come about because of a decrease in the school-age population.

Although career development is an important issue for international students, the topic has received insufficient attention from Korean scholars. Recently, studies have examined educational policies (e.g., Li and Lee, 2014), curriculum development for international students (e.g., Kwon, 2013), and the economic impact on university and nation in recruiting these students (Ha et al., 2015). Some studies (Na, 2006; Park, 2011; Back, 2016; Li et al., 2016) have focused on the need to understand students’ adaptation and acculturation to campus life and the surrounding local community. Few studies have attempted to discover the factors influencing international students’ career development. For example, Heo and Son (2011) found that a lack of career guidance could be a barrier for Chinese international students in Korea. Furthermore, an acceptance of Korean culture (Jiang, 2012) and length of stay (Cui, 2011) were found to be positively related to career development among international students in South Korea. The dearth of information on international students’ career development suggests that further investigation is necessary, and, as such, it was the starting point of the current study.

According to the social cognitive career theory (SCCT), cultural factors affect an individual’s experiences, while these experiences can affect self-efficacy and outcome expectations, which in turn affect an individual’s career choices (Lent et al., 2002). If SCCT was applied to the career development of an international student in South Korea, whether this student endorses their heritage culture or mainstream culture, which stands for Korean culture in this case, might determine their learning experiences, while these experiences can affect their confidence in their ability to function in both cultures, which in turn affects their career development. Therefore, in this study, cultural factors were tested using acculturation, self-efficacy was investigated using bicultural self-efficacy, and career choice was examined through the lens of career decision-making self-efficacy.

Previous studies (Patel et al., 2008; Jiang, 2012; In, 2016) have indicated that acculturation is an important factor in international students’ career decision-making self-efficacy. Career-decision making self-efficacy (CDMSE) refers to an individual’s confidence in making a career decision, which is a strong predictor of career identity and career-related outcome (Betz and Voyten, 1997). In the current study, CDMSE stands for self-efficacy in career decision-making in both countries: international students’ country of origin and South Korea. Acculturation is a process of change that occurs in various domains such as attitude, behavior, values, and identity as a result of continuous and direct contact with individuals of different cultural origins (Redfield et al., 1936; Ryder et al., 2000). More specifically, according to the Mindsponge mechanism, acculturation can be seen as the process of absorbing cultural values into a person’s mindset (which consists of a set of core cultural values) (Vuong and Napier, 2015). Over the years, acculturation has been re-conceptualized in a bidimensional rather than a unidimensional model (Ryder et al., 2000; Miller, 2007). The bidimensional model (Berry, 1997) posits that the two identifications, acculturation to mainstream culture and acculturation to heritage culture, are independent. In the current study, acculturation to mainstream culture refers to acculturation to Korean culture, whereas acculturation to heritage culture refers to acculturation to the international student’s culture of origin.

Previous studies (Patel et al., 2008; Jiang, 2012; In, 2016) have found that the degree of acculturation of international students or ethnic minorities to mainstream culture is related to their career-development process. However, these studies have generated inconsistent results. For instance, English-language acculturation accounted for a unique variance in predicting career decision-making self-efficacy in a group of Vietnamese adolescents living in the United States (Patel et al., 2008). In a sample of Korean international students in the United States, acculturation to mainstream culture was also found to be predictive of career decision-making self-efficacy (In, 2016), and a study (Jiang, 2012) of Chinese international students in South Korea produced similar results. Another study (Wu, 2009) also showed that acculturation to heritage culture is related to career decision-making self-efficacy. Specifically, Wu found that a higher degree of acculturation to heritage culture predicted a higher rate of goal selection, a subscale of career decision-making self-efficacy.

Most prior studies concern international students or racial or ethnic minorities in the United States, and the results have been inconsistent. For example, some studies indicate that acculturation to mainstream culture is related to career development, whereas other studies indicate that acculturation to heritage culture is related to career development. Therefore, the association of both bidimensional acculturation processes with career decision-making self-efficacy should be tested in the South Korean context. In other words, it seems necessary to empirically examine the relationship between each of the two acculturation processes and career decision-making self-efficacy for international students in South Korea.

Bicultural self-efficacy refers to an individual’s subjective belief in their ability to cope with the demands of two different cultural norms—the heritage culture and the host culture. In this study, the definition of bicultural self-efficacy refers to the conceptualization described by LaFromboise et al. (1993) and David et al. (2009). David et al. (2009) proposed six bicultural competencies: (a) knowledge of cultural beliefs and values, (b) positive attitudes toward both cultures, (c) bicultural efficacy, (d) communication ability, (e) role repertoire, and (f) social groundedness. In the current study, an international student with high bicultural self-efficacy might be described as follows: An international student from Mongolia has a strong understanding of both Mongolian and Korean cultural norms and values and has positive feelings toward both countries. The student satisfies the requirements of both cultural norms with a high degree of confidence, is fluent in both languages, exemplifies strong social skills among their Korean and Mongolian peers, and is comfortable networking in both group demographics.

Several empirical studies have found bicultural competence in ethnic minorities to be related to positive psychological functioning. For instance, Wei et al. (2010) reported that higher perceived bicultural competence buffers the association between minority stress and depressive symptoms in students belonging to an ethnic minority in the United States. In addition, Hong (2010) investigated the positive role of bicultural competence in team effectiveness. From these previous studies, bicultural self-efficacy could be a protective facilitator in repelling negative factors, such as depression, while enhancing the positive aspects of a work environment, such as team effectiveness.

Acculturation and bicultural self-efficacy are somewhat related, however, they are two different concepts. Acculturation refers to the extent that one’s attitude, values, and behavior, among other things, allow a person to endorse the heritage or mainstream culture, whereas bicultural self-efficacy implies a perceived confidence to function effectively within both the heritage and mainstream culture. For instance, a person with high acculturation to the mainstream culture would integrate many of the values of the mainstream culture into their core value system (Vuong and Napier, 2015), while a person with high bicultural self-efficacy would feel confident in searching for a job in both Korea and their country of origin. However, very few studies have directly examined bicultural self-efficacy as a positive psychological facilitator of career-related variables for international students in South Korea.

This study is exploratory in nature. International students who possess two cultural heritages might not efficiently develop their career decision-making self-efficacy without being confident in their ability to function well inside or outside of the two cultural groups because information gathering and networking are essential to career development. In other words, it might be that the way international students’ acculturation affects their career decision-making self-efficacy is based on the level of confidence in their ability to function well in both cultures. Therefore, the current study cautiously set out two main exploratory research questions to investigate whether international students’ bicultural self-efficacy would be a mediator between the relationship of acculturation and career decision-making self-efficacy for international students in South Korea:

Question 1: Does bicultural self-efficacy have a meditating effect on the relationship between acculturation to the mainstream culture and career decision-making self-efficacy?
Question 2: Does bicultural self-efficacy have a mediating effect on the relationship between acculturation to the heritage culture and career decision-making self-efficacy?

## Materials and Methods

### Participants

The participants in this study were recruited from Korea University in Seoul, South Korea, through an online survey. The survey was given in Korean because the participants this study targeted were university students and as such were assumed to possess a qualifying Korean-language ability. The researchers who conducted this study received support from the Korea University Career Development Center, which provided a list of 300 international students’ school email addresses. The researchers sent the link to the online questionnaire to the 300 addresses twice, with a 1-week interval in between. All potential participants were treated in accordance with the code of ethics and conduct of the Korean Counseling Association. Participation was entirely voluntary and informed consent was obtained from all participants. Furthermore, the participants were given the researchers’ email addresses and phone numbers to allow them to ask questions related to the research process. Surveys that did not achieve a 90% completion rate were excluded from the analysis and a total of 120 responses were included in the analysis. There were 46 male (38.3%) and 74 female (61.7%) participants with a mean age of 23 years (*SD* = 2.24). The respondents were from 24 countries, with more than half (*n* = 63) from China; 54 (45%) respondents had lived in South Korea for less than 3 years, 59 (49.1%) for 3–10 years, and 7 for “other” (5.8%). The mean level of Korean-language ability for participants was 6.15 (*SD* = 1.01, Range = 1–7), with 7 representing the highest level of Korean-language ability.

### Instruments

The respondents were asked to complete surveys that measured their responses using three scales: a modified version of the Acculturation Scale, the Korean version of the Bicultural Self-Efficacy Scale, and the Korean version of the Career Decision Making Self-Efficacy Scale. At the end of the survey, demographic information (e.g., gender) was also collected.

#### Acculturation

The current study uses the Korean version of the Acculturation Scale (Kim and Kwon, 2009), a validated scale of the Vancouver Index of Acculturation (VIA; Ryder et al., 2000), to measure respondent acculturation. Specifically, the scale measured two subscales: mainstream culture and heritage culture. The scale consists of 20 items and uses a nine-point Likert scale ranging from 1 (*strongly disagree*) to 9 (*strongly agree*). A sample item in the mainstream culture scale states: “I feel comfortable using Korean in front of other people.” A sample item in the heritage culture scale states: “I feel comfortable using my mother tongue in front of other people.” The Cronbach’s alphas for the original scale by Kim and Kwon (2009) were 0.82, 0.80, and 0.63 for acculturation to mainstream culture, acculturation to heritage culture, and total scale, respectively. In the current study, the Cronbach’s alphas were 0.90 and 0.89 for acculturation to mainstream and heritage cultures, respectively. For the validity of the scale, factorial validity was demonstrated by applying Ryder et al. (2000), in which two components were extracted through principal component analysis. Furthermore, in Ryder et al.’s (2000) study, both heritage and mainstream subscales had significant correlations to the Suinn-Lew Asian Self-Identity Acculturation Scale (Suinn et al., 1992), demonstrating this scale’s concurrent validity.

#### Bicultural Self-Efficacy

The Bicultural Self-Efficacy Scale (BSES) was designed to measure an individual’s bicultural self-efficacy (David et al., 2009). The scale consists of 26 items on a nine-point Likert scale, ranging from 1 (*strongly disagree*) to 9 (*strongly agree*). It has the following six dimensions: social groundedness, communication ability, positive attitudes, knowledge, role repertoire, and bicultural beliefs.

Based on the recommendation of Brislin (1986), the current study applied the back-translation technique to validate the BSES. The validation procedure was conducted as follows. First, the BSES was translated into Korean by a bilingual individual enrolled in a master’s program in counseling who was raised in Korea but completed her secondary and postsecondary education in the United States. Then, three doctoral students majoring in counseling reviewed and modified the translated version. The translated version was then back-translated into English by another bilingual individual who had not read the original version. This bilingual individual was a Korean-Canadian enrolled in the same master’s program as the first translator. Then, the researchers compared the original version of the BSES and the back-translated version to identify any discrepancies and modified a few words and phrases. Afterward, the back-translated version of the BSES was reviewed by the original author. The final revision of the Korean version of the BSES was produced based on feedback from two professors who are experts on international students.

The Cronbach’s alphas for the original bicultural self-efficacy scale by David et al. (2009) and the Korean version were 0.94 and 0.98, respectively. For the validity of this scale, factorial validity was demonstrated using the study conducted by David et al. (2009), in which the six factors proved to be the most appropriate structure for measuring bicultural self-efficacy. However, the current study suggests that the unidimensional model would be suitable for the data used in this study. Furthermore, in the study by David et al. (2009), the construct validity of the BSES was supported by the results showing a positive correlation between bicultural self-efficacy and acculturation and enculturation and no correlation between BSES and social desirability.

#### Career Decision-Making Self-Efficacy

This study used the Korean version of the Career Decision-Making Self-Efficacy Scale—Short Form (CDMSE-SF; Lee, 2001), a validated translation of Betz and Voyten’s (1997) CDMSE-SF. Specifically, the CDMSE-SF measures five subscales: self-appraisal (e.g., determine what your ideal job would be), occupational information gathering (e.g., find information in the library), goal selection (e.g., select one college major from a list of potential majors you are considering), planning (e.g., make a plan for your goals for the next 5 years), and problem solving (e.g., continue to work on your major or career goal even when you become frustrated). However, for CDMSE-SF, using Rasch’s rigorous model, Nam et al. (2011) reported that the single-factor solution of CDMSE-SF was the best fit in the Korean context (Nam et al., 2011). It consists of 25 items and uses a five-point Likert scale (1 = *strongly disagree* to 5 = *strongly agree*). The Cronbach’s alphas for the original scale range from 0.69 to 0.83, and that for the total CDMSE-SF is 0.93. The Cronbach’s alphas for the current study are 0.79 for self-appraisal, 0.72 for gathering occupational information, 0.75 for goal selection, 0.77 for goal identification, and 0.64 for problem solving. The Cronbach’s alpha was 0.93 for the five-item CDMSE-SF used in this study. For the validity of this scale, Betz and Taylor (1994) provided the original 50-item version of the CDMSE-SF in its manual, and Betz and Voyten (1997) reported that the CDMSE-SF is positively associated with exploratory intentions and decreased career indecision as assessed by the Career Decision Scale (CDS; Osipow, 1987). As for Korean university students, Lee and Lee (2000) found a correlation between the CDMSE-SF and CDS, which provided the criterion validity for the CDMSE-SF scale.

### Data Analysis

The data were analyzed using structural equation modeling (SEM) with Amos 21.0 (Kline, 2015). First, a full measurement model was specified, and confirmatory factor analysis (CFA) was conducted to test the appropriateness of the current data. The item parceling technique was used to construct the observed variables (bicultural self-efficacy and career decision-making self-efficacy) in the measurement model for following reasons. First, according to the evidence collected, bicultural self-efficacy is unidimensional in the Korean context (Lee and Lim, 2015; Lee and Park, 2019), rather than being organized into the six-factor structure proposed by its original developers David et al. (2009). In addition, although career decision-making self-efficacy is well represented by five subscales in the Western context, there is evidence that a unidimensional construct is preferable in the Korean context (Nam et al., 2011). Second, parceling could provide more stable factor solutions than item-level data (Little et al., 2002).

According to Little et al. (2002), the item-parceling technique is a measurement practice that can be used in multivariate approaches; and generally, a parcel is an aggregate-level indicator consisting of the sum (or average) of two or more items. Specifically, nine parcels (i.e., four for measuring bicultural self-efficacy and five for measuring career decision-making self-efficacy) were created for this study. In addition, each parcel was created by calculating the average of a set of five to seven items that were randomly assigned to each parcel.

Second, a structural model was specified to test the hypothesized mediation model. To decide the model fit for both the measurement and structural models, fit indices, including the Comparative Fit Index (CFI), Tucker-Lewis Index (TLI), Root Mean Square Error of Approximation (RMSEA), and Standardized Root Mean Square Residual (SRMR), were used with cutoff values recommended by Hu and Bentler (1999). Finally, a bootstrap analysis was performed to examine whether the indirect effects were significant (Shrout and Bolger, 2002). Bootstrapping is a method that uses repeated sampling from a dataset to create a new dataset that is suitable for testing the statistical significance of an indirect effect (Preacher and Hayes, 2008). The current study generated 1,000 bootstrap samples. According to Shrout and Bolger (2002), if the 95% confidence interval (CI) does not include zero, the indirect (i.e., mediation) effect is significant at the 0.05 level.

## Results

The mean, standard deviation, and correlation among the variables are presented in Table 1. Korean-language skills are significantly related to most variables but not to acculturation to heritage culture. However, length of stay does not correlate to most of the measured variables. Acculturation to heritage culture, acculturation to mainstream culture, bicultural self-efficacy, and career decision-making self-efficacy were positively correlated with each other, with correlations among the variables ranging from *r* = 0.31 (*p* < 0.01) to *r* = 0.75 (*p* < 0.01).

**TABLE 1:** Means, standard deviations, and correlations among variables.

	*M*	*SD*	1	2	3	4	5	6
1. Korean	6.15	1.02	–					
2. Length of Stay	4.63	3.84	0.31**	–				
3. ACC_H	6.00	1.42	0.17	–0.09	–			
4. ACC_M	5.95	1.35	0.34**	0.09	0.61**	–		
5. BSES	6.15	1.39	0.44**	0.16	0.45**	0.75**	–	
6. CDSE	3.23	0.60	0.28**	0.09	0.31**	0.41**	0.49**	–

**p < 0.01, N = 120, M, mean; SD, standard deviation; Korean, Level of Korean; Level of Korean: 1 = low, 7 = high; ACC_H, acculturation to heritage culture; ACC_M, acculturation to mainstream culture; BSES, bicultural self-efficacy; CDSE, career decision-making self-efficacy.

**FIGURE 1:**
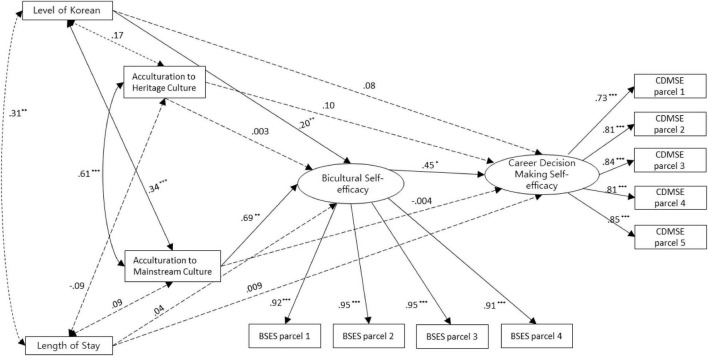
Research design of the structural model. ***p < 0.001, **p < 0.01, and *p < 0.05.

Next, prior to examining the structural model, a full measurement model was created to test the appropriateness of the current data. The model fit of the full measurement model was acceptable according to Hu and Bentler’s (1999) criteria: χ^2^(54) = 99.16, *p* = 0.00, CFI = 0.96, TLI = 0.95, RMSEA = 0.08. The factor loadings for the measurement model are presented in Table 2. In addition, the structural model was then examined to assess whether bicultural self-efficacy has a mediating effect on the relationship between acculturation to heritage culture and career decision-making self-efficacy simultaneously with that between acculturation to mainstream culture and career decision-making. Korean-language skills and length of stay are included in the mediation model as control variables. Several previous studies (e.g., Patel et al., 2008; Cui, 2011) reported that these two variables affect many aspects related to career development. The research design of the structural model is presented in Figure 1. The model fit of the structural model was acceptable: χ^2^(54) = 99.16, *p* = 0.00, CFI = 0.96, TLI = 0.95, RMSEA = 0.08, indicating that the model was fit for the current data.

**TABLE 2:** Factor loadings for the measurement model (*N* = 120).

Measure and variable	Unstandardized factor loading	*SE*	Z	Standardized factor loading
**Bicultural self-efficacy**				
Bicultural Self-Efficacy parcel 1	0.95			0.92***
Bicultural Self-Efficacy parcel 2	0.99	0.06	16.89	0.95***
Bicultural Self-Efficacy parcel 3	1.09	0.05	19.03	0.95***
Bicultural Self-Efficacy parcel 4	1.00	0.06	18.64	0.91***
**Career decision-making self-efficacy**				
Career Decision-Making Self-Efficacy parcel 1	1.00			0.73***
Career Decision-Making Self-Efficacy parcel 2	1.15	0.13	8.68	0.81***
Career Decision-Making Self-Efficacy parcel 3	1.11	0.12	9.05	0.84***
Career Decision-Making Self-Efficacy parcel 4	1.09	0.13	8.73	0.81***
Career Decision-Making Self-Efficacy parcel 5	1.19	0.13	9.16	0.85***

***p < 0.001.

The path coefficients (ß = 0.69, *p* < 0.01) from acculturation to mainstream culture to bicultural self-efficacy and those *(ß* = 0.45, *p* < 0.05) from bicultural self-efficacy to career decision-making self-efficacy were statistically significant. However, the path coefficient (ß = −0.004) from acculturation to mainstream culture to career decision-making self-efficacy was not significant. In addition, the path coefficients from acculturation to heritage culture to bicultural self-efficacy and those from acculturation to heritage culture to career decision-making self-efficacy were non-significant. Only the path from bicultural self-efficacy to career decision-making was significant (ß = 0.45, *p* < 0.05). In addition, acculturation to heritage culture and acculturation to mainstream culture were highly related (ß = 0.61, *p* < 0.001).

Finally, a bootstrap was performed to test the indirect effect of the mediation model, which examined whether bicultural self-efficacy had a mediating effect on acculturation to mainstream culture and career decision-making self-efficacy. According to Shrout and Bolger (2002), if the 95% CI does not include zero, the indirect effect can be considered significant at the 0.05 level. The results indicated that acculturation to mainstream culture had a significant indirect effect on career decision-making self-efficacy [95% CI = (0.103–0.542), *b* = 0.12, ß = 0.31]. Since the path between acculturation to mainstream culture and career decision-making self-efficacy was not significant, it could be said that bicultural self-efficacy fully mediated the relationship between these two variables.

## Discussion

The current study examined whether bicultural self-efficacy has a mediating effect on the relationship between acculturation to mainstream or heritage culture and career decision-making self-efficacy. The results indicate that bicultural self-efficacy fully mediated the relationship between acculturation to mainstream culture and career decision-making self-efficacy, thus Research Question 1 was resolved. However, bicultural self-efficacy did not have a mediating effect on the relationship between acculturation to heritage culture and career decision-making self-efficacy (Research Question 2).

In previous empirical studies (Patel et al., 2008; Jiang, 2012; In, 2016), acculturation to mainstream culture was positively correlated to career decision-making self-efficacy. However, in the current study, when bicultural self-efficacy was added as a mediating variable, the direct relationship between acculturation to mainstream culture and career decision-making self-efficacy disappeared, revealing that acculturation to mainstream culture likely has an indirect effect on career decision-making self-efficacy *via* bicultural self-efficacy.

For the international students in the current study, maintaining a high level of acculturation to mainstream culture—including being fluent in Korean, understanding Korean cultural norms, and acclimating to the Korean diet—could lead to high bicultural self-efficacy and, subsequently, to high career decision-making self-efficacy. This implies that acculturation to mainstream culture (Korean culture) is more important than acculturation to heritage culture in determining international students’ career decision-making self-efficacy. The results also indicate that acculturation to mainstream culture by itself may not facilitate international students’ career development, but could if coupled with bicultural self-efficacy. These results extend SCCT in the Korean context.

Wu (2009) reported that international students’ acculturation to the heritage culture was related to the goal-selection subscale of career decision-making self-efficacy. The current results are inconsistent with those of previous studies in that no significant relationship was observed between acculturation to heritage culture and career decision-making self-efficacy, and the relationship between acculturation to heritage culture and bicultural self-efficacy was not significant. This suggests that improving acculturation to heritage culture may not directly improve international students’ bicultural self-efficacy and career decision-making self-efficacy. In addition, In (2016) found a correlation between acculturation to heritage culture and career decision-making self-efficacy, but when other variables such as Hope were added, the significant relationship between acculturation to heritage culture and career decision-making self-efficacy disappeared. This result indicates that acculturation to heritage culture may improve or inhibit career decision-making self-efficacy if incorporated with other variables. However, which variables can interfere with acculturation to heritage culture should be studied further in future research.

Regarding international students’ career decision-making self-efficacy, this study suggests that improving acculturation to mainstream culture and bicultural self-efficacy are more effective than focusing on acculturation to heritage culture. For example, Chinese international students who spend most of their time with their Chinese peers and do not actively involve themselves in learning Korean culture, language, or norms, are likely to have greater difficulty in maintaining high career decision-making self-efficacy. A more efficient way to improve career decision-making self-efficacy could involve interactions with both Korean and Chinese colleagues and developing bicultural competence.

The results of the current study could be examined through the Mindsponge mechanism (Vuong and Napier, 2015), which is a multi-filtering information process. The Mindsponge mechanism assumes that every person has a mindset, or a set of core cultural values or beliefs, and proposes that acculturation is a process of absorbing and ejecting cultural values using the person’s core values as benchmarks. Through the lens of the Mindsponge mechanism, if an international student possessed more Korean cultural values, they would be better able to navigate their career options in both cultures, especially given that a lot of international students do work in fields related to Korea. More specifically, if international students attained more Korean cultural values (*via* acculturation to mainstream culture), then they would be able to expand the amount of Korean cultural knowledge in their mindset. Acquiring more Korean cultural knowledge could improve their perceived familiarity with the Korean culture and environment and give them more competitiveness than students who are only equipped with heritage cultural knowledge. Furthermore, cultural familiarity and competitiveness could lead them to have high confidence in their ability to function appropriately in both cultural situations, which in turn would help them to attain more information related to their career development and give them greater self-efficacy in the career decision-making process.

The results of the current study could also be partially explained by the social and cultural environment of Korea and the Korean job market. Although the immigrant population, which includes migrant workers, marriage migrants, and international students, has prompted Korea to begin transitioning from a monoethnic, culturally homogeneous society to a multiethnic, multicultural one, the process is still in the early stages of development. The Korean government has implemented policies to help newcomers assimilate to the host country, but these policies focus more on language learning and cultural education than on helping them integrate their existing culture with their new one or on addressing issues of ethnic, racial, and cultural diversity. To develop career-related competence in the Korean context, it is likely that international students will need to be equipped with an understanding of Korean culture.

In addition, many international students prefer to seek employment in Korea after they finish their degree, and most seek to acquire 2–3 years of work experience in Korea. Of those who return to their home country, most work in Korean companies or do work that is related to Korea. Students searching for a job will almost certainly need to interact with Korean nationals, and they may need to follow Korean cultural norms when searching for a job (e.g., prepare a Korean-style résumé, dress professionally according to Korean work customs, become familiar with Korean interview etiquette), all of which could put pressure on the job seeker and impact their career development. In particular, international students gather information on job searching and seek employment referrals from Korean friends; therefore, understanding Korean culture and having strong social networks among Koreans is especially important in developing career decision-making self-efficacy.

The current results produce the following practical implications for the career development of international students. University career counseling centers could play an important role by training on-campus career counselors in issues related to biculturalism or multiculturalism. The career counseling training curriculum may also need to incorporate culture-focused career development theories, such as the four strategies for coping with acculturation (assimilation, integration, separation, and marginalization) proposed by Berry (1997) and the six components of bicultural self-efficacy (LaFromboise et al., 1993; David et al., 2009).

When providing career counseling to international students, career counselors may need not only to practice traditional career development theory, they may also need to consider the individual’s level of acculturation and bicultural efficacy. Career counselors can assess a student’s bicultural self-efficacy with the Bicultural Self-Efficacy Scale (BSES) and/or the Vancouver Index of Acculturation (VIA), and can use the results to create and implement educational intervention programs designed to improve students’ bicultural self-efficacy and level of acculturation to mainstream culture.

Career development courses that introduce or examine the concept of biculturalism could be particularly beneficial for international students. By acknowledging the importance of biculturalism in their career development, international students might recognize that valuing both cultures could greatly improve their career decision-making self-efficacy. Educational intervention programs could focus on providing international students with practical information about the job-search process in Korea, including where to find recruiting information, how to write a Korean résumé, job interview etiquette, and Korean business attire. Other programs could introduce students to Korean company culture, office etiquette, and business practices. Counselors could also design mentoring programs that connect international students with Korean students so they can build academic and professional networks to exchange information.

The current study has the following limitations. First, the sample size is relatively small and 60% of the participants are from China, so caution is needed when generalizing the results. In future studies, it might be helpful to examine the relationship between cultural factors and career variables separately according to the country of origin. Second, the average Korean-language level of the participants in the current study is quite high with an average of 6 out of 7. This is because the participants were recruited from one of the top-ranked universities in South Korea, which has relatively high admission requirements regarding Korean-language ability. In addition, because students with a high level of acculturation to Korean culture were more likely to participate and those with a low level of acculturation to Korean culture are largely absent from the sample, the relationships among the variables (i.e., correlation coefficients) might be under or overestimated (Rohrer, 2018). Thus, caution will be needed in generalizing the results of the current study and the samples must be diversified in future studies.

Third, the current study confirmed the effectiveness of bicultural self-efficacy in theory, but future study will be needed to develop specific educational intervention programs using the bicultural self-efficacy model and to confirm the effectiveness of the programs in a real setting. Fourth, the current study using bicultural self-efficacy scales did not go through a validation process; therefore, in future studies, cross-validation of BSES is suggested.

Fifth, the effects of bicultural self-efficacy and general self-efficacy on career decision making self-efficacy may overlap. Therefore, in future studies, these two variables should be tested separately. Finally, the role of bicultural self-efficacy in acculturation and career decision making self-efficacy may differ in terms of where job-search activities take place. Therefore, in future studies, the model proposed in this study should be tested by separating international students who are searching for jobs in South Korea and those searching in their home countries.

The current study empirically investigated the effects of cultural factors on career development in the Korean context, and the results of the current study extend SCCT in the Korean context. More specifically, the current study confirmed the effect of acculturation to mainstream culture and the effect of bicultural self-efficacy on career development. In addition, the current study found that compared to acculturation to heritage culture, acculturation to mainstream (Korean) culture is the most significant factor in career development for international students in Korea. In summary, the current results indicate that career counselors should fully consider international students’ cultural context when designing and implementing career counseling programs.

## Data Availability Statement

The raw data supporting the conclusions of this article will be made available by the authors, without undue reservation.

## Ethics Statement

The studies involving human participants were reviewed and approved by IRB of Korea University. The patients/participants provided their written informed consent to participate in this study.

## Author Contributions

All authors listed have made a substantial, direct, and intellectual contribution to the work, and approved it for publication.

## Conflict of Interest

The authors declare that the research was conducted in the absence of any commercial or financial relationships that could be construed as a potential conflict of interest.

## Publisher’s Note

All claims expressed in this article are solely those of the authors and do not necessarily represent those of their affiliated organizations, or those of the publisher, the editors and the reviewers. Any product that may be evaluated in this article, or claim that may be made by its manufacturer, is not guaranteed or endorsed by the publisher.
